# Resolution of Vulvar Lichen Sclerosus Symptoms Following Dietary Elimination of Tomato and Nightshade Vegetables: A Case Report

**DOI:** 10.7759/cureus.91862

**Published:** 2025-09-08

**Authors:** Jill Hutton

**Affiliations:** 1 Obstetrics and Gynecology, Hormonal Well-Being, Bellaire, USA

**Keywords:** allergy to food, female genital tract, igg antibodies, lichen sclerosus, non-ige mediated, vulvar dermatoses, vulvar lichen sclerosus

## Abstract

Lichen sclerosus (LS) is a chronic inflammatory skin condition that most often affects the perineal region and can greatly impact quality of life. This report presents the case of a 69-year-old woman with a 20-year history of vulvar LS. Despite standard treatment with topical corticosteroids, as well as multiple other therapies, including some nonstandard approaches, her symptoms persisted. Laboratory evaluation revealed elevated IgG antibodies to tomato. After eliminating tomatoes and other nightshade vegetables from her diet, the patient remained symptom-free for over a year. This case highlights a potential role of dietary antigens in LS and underscores the need for further investigation of non-IgE-mediated food sensitivities in managing symptoms.

## Introduction

Lichen sclerosus (LS) is a chronic inflammatory skin disorder characterized clinically by thin, pale, patchy skin that may be itchy, painful, or prone to tearing. Symptoms may fluctuate, with periods of remission and flare-ups. Over time, affected areas may broaden with the skin becoming hypopigmented and scarred. About 85% of cases involve the anogenital region [1]. The incidence is estimated at 0.1-0.3% of the population, with women affected more often than men at a ratio ranging from 3:1 to 10:1. Beyond local discomfort, LS can cause pain during intercourse, frequently leading to avoidance. It may also cause discomfort during urination and, less commonly, bowel movements. The exact cause remains unknown, though autoimmune mechanisms are believed to play a role. Additional associations have been reported with hormonal influences, trauma, and genetic predisposition [1]. If left untreated, LS can result in scarring and, in rare cases, an increased risk of skin cancer. Treatment typically involves mid- to high-potency topical corticosteroids to reduce inflammation and manage symptoms [1,2].

This report describes a 69-year-old woman with a 20-year history of vulvar LS. Despite standard treatment with topical corticosteroids, as well as multiple other therapies, her symptoms persisted. Laboratory testing revealed elevated IgG antibodies to tomato. After eliminating tomatoes and other nightshade vegetables from her diet, she remained symptom-free for over a year. This case suggests a potential role of dietary antigens in LS and encourages the need for further exploration of non-IgE-mediated food sensitivities in symptom management.

## Case presentation

A rather healthy 69-year-old postmenopausal woman presented with chronic vulvar irritation and pruritus persisting for nearly two decades. She is married, gravida 2, para 2, postmenopausal, and underwent a hysterectomy at age 55 with ovarian conservation. She initiated estrogen replacement therapy shortly after surgery and continues to use it. Initially, she took conjugated equine estrogens for approximately 18 months, then switched to estradiol and added bedtime micronized progesterone. In this case, progesterone was not required for endometrial protection, but added for its neuroactive properties, including modulation of gamma-aminobutyric acid (GABA) receptors, which can confer anxiolytic effects and improve sleep quality [3]. Her medical history includes mild gastroesophageal reflux disorder and chronic constipation, both evaluated twice with endoscopy and colonoscopy, yielding negative findings. At 61 years, she had a right hemithyroidectomy for a benign hyperplastic nodule, followed by extensive endocrine testing, which was unremarkable except for hypothyroidism. She is treated with both levothyroxine and liothyronine. At age 68, she had cataract surgery and was diagnosed with dyslipidemia, for which she takes ezetimibe. She is a lifelong non-smoker and reports no other significant social habits.

Her symptoms of intense itching in the vulvar and rectal areas, perineal and rectal pressure, and avoidance of intercourse began in her late 40s. She describes an irritation in the perineal area that is raw from scratching all night. In search of relief, she tried numerous measures: switching soaps, toilet paper, bed sheets, and laundry detergents; testing her water; avoiding pajamas and underwear; trying different styles of undergarments; applying petroleum jelly and olive oil; taking magnesium sulfate baths; avoiding baths altogether; using different towels; and eliminating plastic food containers. Despite these efforts, she continued to experience significant discomfort and anxiety, knowing each night she was unlikely to achieve peaceful sleep.

A biopsy at the time confirmed LS, and she was intermittently treated with topical steroids such as clobetasol 0.05% cream as prescribed. Once she was given an injection of clobetasol for a more long-lasting steroid effect, it gave her palpitations. She also used hydrocortisone in varying strengths, localized estradiol cream, and lidocaine and/or prilocaine creams as needed. She tried oral cromolyn and antihistamines, but these made her feel “wired.” Seeking alternative treatments, she completed multiple courses of antifungals to eliminate yeast, as well as of valacyclovir to target any “latent virus.” Her LS also caused persistent sleep difficulties, for which she frequently used melatonin, magnesium, and zolpidem. Despite these interventions and multiple consultations over the years, her symptoms persisted.

**Figure 1:**
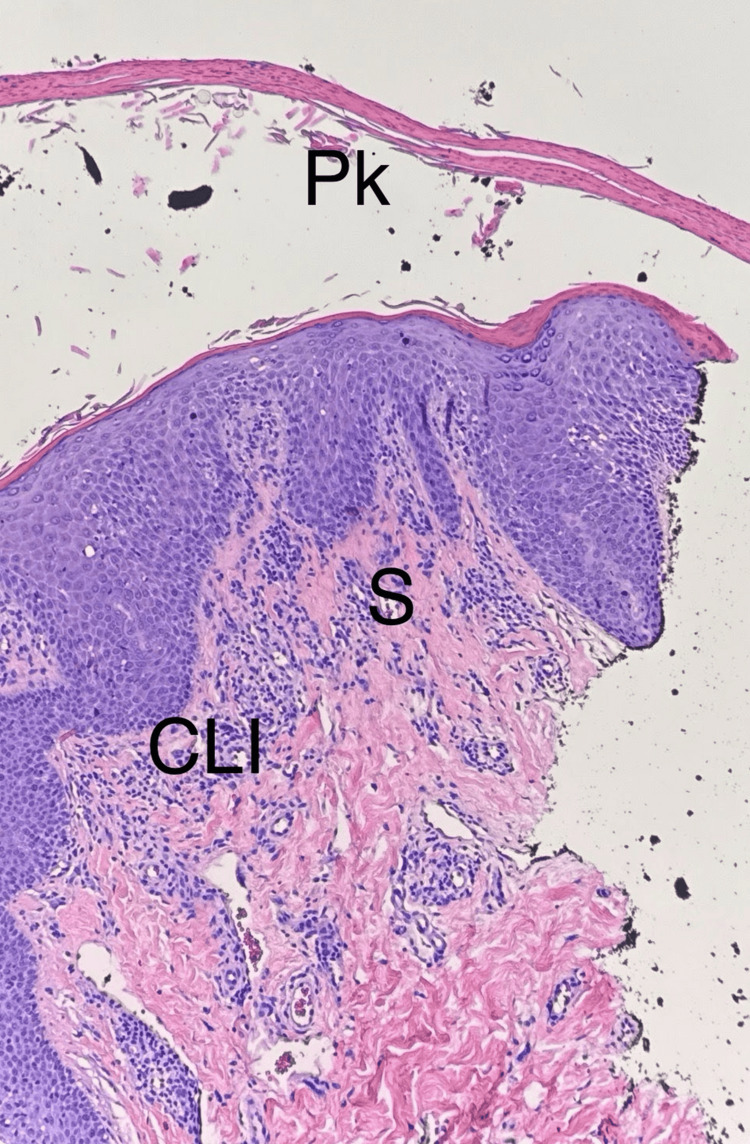
Biopsy showing chronic lichenoid inflammation (CLI), sclerosis (S) and parakeratosis (Pk)

At age 67, reevaluation showed mild vaginal and urethral atrophy, broad areas of depigmentation, and a well-demarcated 3-4 centimeter hypopigmented plaque on the left labia. Erythema was noted, but no notable concurrent excoriations. A repeat 4 mm punch biopsy (Figure 1) of the left labia demonstrated chronic dermatitis with underlying lichenoid tissue, supporting the clinical diagnosis of LS. She reported no known allergies and was generally healthy, with hypothyroidism and hypercholesterolemia managed medically. Routine laboratory results were unremarkable.

The same year, she experienced a wasp sting that caused facial swelling. She presented to the emergency department, where she received epinephrine and IV solumedrol, and was subsequently referred to an allergy specialist. At age 68, laboratory testing showed a white blood cell count of 4.18 K/µL with normal indices, including eosinophils at 1.9% (69 cells/µL). She was confirmed to be allergic to wasp venom and now carries an epinephrine auto-injector. Additional allergy testing (Table 1) revealed negative IgE responses to celery, lettuce, orange, parsley, and tomato. However, IgG antibodies to tomato were elevated at 3.6 µg/mL (reference upper limit: 2.0 µg/mL).

**Table 1:** Allergen-specific IgE and IgG antibody results

Analyte	Value	Reference Range	Units	Abnormal Flag
Celery (F85) IgE	<0.10	<0.10, absent/undetectable	kU/L	Normal
Lettuce (F215) IgE	<0.10	<0.10, absent/undetectable	kU/L	Normal
Orange (F33) IgE	<0.10	<0.10, absent/undetectable	kU/L	Normal
Parsley (F86) IgE	<0.10	<0.10, absent/undetectable	kU/L	Normal
Tomato (F25) IgE	<0.10	<0.10, absent/undetectable	kU/L	Normal
Tomato (F25) IgG	3.6	<2.0	mcg/mL	High

Based on these findings, dietary elimination of tomatoes and other nightshade vegetables, most notably peppers, potatoes, and eggplants, was advised. During the sequential elimination process, she noted symptom flares of her LS symptoms after consuming salsa, which typically contains tomatoes, onion, garlic, cilantro, jalapeno pepper, and lime juice. After removing tomatoes and peppers from her diet, she reported complete resolution of symptoms. Improvement occurred within approximately two weeks and has been sustained for over one year.

## Discussion

The exact cause of LS remains unclear; however, hormonal factors have been implicated, as the condition is often diagnosed before puberty and after menopause. Other possible contributors include chronic irritation or trauma and autoimmune conditions [1,2], particularly autoimmune thyroid disorders. Approximately 12% of individuals with LS have either Hashimoto’s thyroiditis or Graves’ disease [4]. In this case, although the patient has hypothyroidism, she has not been diagnosed with Hashimoto’s or Graves’ disease. Her LS diagnosis preceded menopause, and she reported frequent irritation, itching, and discomfort.

While LS is not traditionally linked to food sensitivity, this case suggests a possible role for non-IgE-mediated immune responses in symptom persistence. Tomato allergy is among the most common vegetable allergies in Europe, with an estimated prevalence of approximately 4.9%, particularly higher in southern regions such as Spain and Italy [5]. It can present as either an IgE-mediated or a non-IgE-mediated reaction. The IgE-mediated form is typically more acute and severe, ranging from oral allergy syndrome - characterized by irritation of the lips, oral mucosa, and esophagus - to angioedema and, in rare cases, anaphylaxis. These symptoms typically appear shortly after consuming tomatoes [5].

In contrast, non-IgE-mediated tomato allergy can be more difficult to diagnose, as symptoms are often nonspecific and may not appear until several days after exposure [5,6]. Among patients with tomato-specific IgG antibodies, approximately 75% report skin-related symptoms/conditions (including eczema, psoriasis, dermatitis, acne, hair loss), which appear more frequently than gastrointestinal complaints (self-reported abdominal bloating, heartburn, nausea, and gastritis) [6]. The role of IgG antibodies in food allergies remains controversial, and many allergy societies and physicians do not recommend routine IgG testing as a standard practice. While some literature suggests IgG responses may reflect normal exposure or tolerance, emerging evidence implicates these antibodies in the pathogenesis of conditions such as eosinophilic esophagitis, eczema, and inflammatory bowel disease [7-9]. Notably, among patients with atopic dermatitis who adopted elimination diets, approximately 50% reported symptomatic improvement upon the exclusion of nightshade vegetables [10]. In infants, tomato allergy is a recognized cause of diaper dermatitis [11]. These findings illustrate how IgG antibodies to certain foods may contribute to dermatologic conditions and how targeted food avoidance may address the underlying cause.

In the context of our patient, who experienced frequent irritation, tomato sensitivity appears to be a relevant contributing factor. While standard allergy testing can help identify common allergens, current panels may not include all tomato-derived proteins, potentially resulting in false negatives [5,12]. Therefore, an empirical trial of dietary elimination, specifically avoiding tomatoes and potentially other nightshade vegetables, for eight weeks could be considered a low-risk, diagnostic, and therapeutic approach.

## Conclusions

LS is an inflammatory dermatosis of unknown cause, with symptoms that, as this case demonstrates, can significantly impair quality of life. Food elimination has been shown to improve various dermatological conditions, including psoriasis, eczema, dermatitis, and acne, and may also benefit LS. This case describes a novel association between LS and a suspected non-IgE-mediated allergy to tomatoes, highlighting the potential role of dietary triggers in dermatologic disease. Notably, the patient experienced complete symptom resolution after eliminating tomatoes and other nightshade vegetables, including all varieties of peppers, from her diet. While this represents a single case, it underscores the need for further research into the role of non-IgE-mediated food sensitivities in the pathogenesis and management of LS. Testing for food allergies with both IgE and IgG may reveal previously unrecognized triggers. However, even if test results are negative, an empirical trial of food avoidance, particularly of nightshade vegetables, for eight weeks may be a low-risk strategy worth considering.
